# Deletion of the Mineralocorticoid Receptor in Myeloid Cells Attenuates Central Nervous System Autoimmunity

**DOI:** 10.3389/fimmu.2017.01319

**Published:** 2017-10-13

**Authors:** Elena Montes-Cobos, Nils Schweingruber, Xiao Li, Henrike J. Fischer, Holger M. Reichardt, Fred Lühder

**Affiliations:** ^1^Institute for Cellular and Molecular Immunology, University Medical Center Goettingen, Goettingen, Germany; ^2^Institute of Neuroimmunology, University Medical Center Goettingen, Goettingen, Germany; ^3^Institute for Multiple Sclerosis Research, University Medical Center Goettingen, Goettingen, Germany

**Keywords:** mineralocorticoid receptor, experimental autoimmune encephalomyelitis, myeloid cells, M2 polarization, neuroinflammation

## Abstract

Myeloid cells play an important role in the pathogenesis of multiple sclerosis (MS) and its animal model experimental autoimmune encephalomyelitis (EAE). Monocytes, macrophages, and microglia can adopt two distinct phenotypes, with M1-polarized cells being more related to inflammation and autoimmunity while M2-polarized cells contribute to tissue repair and anti-inflammatory processes. Here, we show that deletion of the mineralocorticoid receptor (MR) in bone marrow-derived macrophages and peritoneal macrophages caused their polarization toward the M2 phenotype with its distinct gene expression, altered phagocytic and migratory properties, and dampened NO production. After induction of EAE, mice that are selectively devoid of the MR in their myeloid cells (MR^lysM^ mice) showed diminished clinical symptoms and ameliorated histological hallmarks of neuroinflammation. T cells in peripheral lymphoid organs of these mice produced less pro-inflammatory cytokines while their proliferation and the abundance of regulatory T cells were unaltered. The numbers of inflammatory monocytes and reactive microglia in the central nervous system (CNS) in MR^lysM^ mice were significantly lower and they adopted an M2-polarized phenotype based on their gene expression profile, presumably explaining the ameliorated neuroinflammation. Our results indicate that the MR in myeloid cells plays a critical role for CNS autoimmunity, providing a rational to interfere with diseases such as MS by pharmacologically targeting this receptor.

## Introduction

Multiple sclerosis (MS) is an inflammatory disease of the central nervous system (CNS) with significant socio-economic relevance, most often affecting young adults. Acute relapses are generally treated by high-dose methylprednisolone (MP) pulse therapy (1, 2), whereas interferon-β products and glatiramer acetate are used to reduce relapse frequency and to slow down long-term progression of the disease (3). More recently, new therapeutic approaches with activity in MS patients have been developed, such as monoclonal antibodies targeting CD20, CD25, CD52, or α4 integrin, and small molecule compounds such as fingolimod, demethyl fumarate, and laquinimod (3). While these new therapeutics have considerably improved the management of MS, they bring with them adverse effects that at least partially constrain their use. For instance, application of the anti-CD52 antibody Alemtuzumab can cause autoimmune diseases such as Hashimoto thyroiditis, whereas therapy with the anti-α4 integrin antibody Natalizumab is associated with an increased risk of progressive multifocal leukoencephalopathy. It is against this background that research aimed at identifying drug targets suitable for MS therapy is still warranted. One promising candidate is the mineralocorticoid receptor (MR), also known as NR3C2 (4). It is a member of the nuclear receptor superfamily, plays a significant role in the regulation of the immune system, and can be targeted by clinically approved compounds that have been in use for many years (5).

The MR resides in the cytosol in the form of a multimeric protein complex and translocates into the nucleus after ligand-binding. It functions as a transcription factor and can activate a large set of genes (6). In many tissues such as in kidney and colon, aldosterone is the major ligand of the MR. Additional binding partners are glucocorticoids (GC), which can bind to the MR with comparable affinity and are present in the circulation at a much higher concentration (7). GC are, therefore, relevant ligands of the MR in cell types which do not express 11β-hydroxysteroid dehydrogenase type 2 (11β-HSD2), an enzyme that can inactivate GC and thereby prevent their action. Myeloid cells are an example of cells in which the MR is almost exclusively bound by GC rather than aldosterone (8). Since myeloid cells not only express the MR but also express the glucocorticoid receptor (GR), activities of corticosterone, cortisol, and synthetic GC in these cells are mediated in a balanced manner by both the MR and GR. For instance, it has been reported that the MR in myeloid cells fosters their polarization toward the M1 phenotype, thus promoting inflammation and autoimmunity, whereas GC acting *via* the GR rather induce a commitment of myeloid cells to the M2 phenotype and thereby support the resolution of inflammation and tissue repair (9). Furthermore, a coordinate control of NF-κB activity and the production of pro-inflammatory mediators by GC *via* the GR and MR were demonstrated in microglia cells (10), further highlighting an important role of the MR in regulating inflammatory processes in different myeloid cell types.

To understand the mechanisms of GC in the control of MS, a number of studies have been conducted in experimental autoimmune encephalomyelitis (EAE), a widely used animal model of this disease [reviewed in Ref. (11)]. GC are well known for their potent anti-inflammatory activities in MS. In T cells, they diminish the level of pro-inflammatory cytokines, downregulate adhesion molecules, induce lymphocyte apoptosis, and reduce the migration of autoreactive T cells into the CNS (12–14). In myeloid cells such as macrophages, GC reduce surface expression of MHC class II molecules, interfere with antigen-presentation, inhibit NO production, and lead to an upregulation of scavenger receptors such as CD163 (15). As of yet, most studies on GC action in neuroinflammatory diseases have focused on the role of the GR. Thus, it has been found that the GR in T cells was critical for the modulation of EAE by free and in part also by liposome-encapsulated GC (12, 15), whereas inorganic–organic hybrid nanoparticles containing betamethasone preferentially targeted myeloid cells and ameliorated EAE even without direct suppression of T cell function (16). By contrast, there is little known concerning the contribution of the MR in mediating GC effects in MS and EAE. In one study, application of the MR agonist deoxycorticosterone acetate was found to aggravate EAE, which could be prevented using the MR antagonist spironolactone (17). Furthermore, a reduction of MR expression in whole blood cells was found in MS patients (18). While these findings indicate a potential involvement of the MR in modulating neuroinflammatory diseases, there have been very few studies addressing this topic overall.

It is generally assumed that the major physiological role of the MR is the regulation of the salt-water balance and various cardiac functions. For example, an ubiquitous inactivation of the MR or its conditional ablation in kidney or cardiomyocytes altered the control of sodium reabsorption and blood pressure, and partially protected from cardiac failure (19–22). Furthermore, specific deletion of the MR in myeloid cells was also found to protect against cardiac hypertrophy, fibrosis, and heart failure, suggesting that the MR in immune cells is more important than previously supposed (9). Such a notion is also supported by the finding that the infarct volume after middle cerebral artery occlusion as an animal model of stroke was reduced in these mutant mice (23). More recently, a critical role of the MR in T cells has been unraveled by demonstrating its involvement in the control of blood pressure and heart pathophysiology (24, 25). Taken together, all these observations provided hints that the MR in myeloid and other immune cells contributes to the regulation of inflammation-associated processes and possibly autoimmune diseases of the CNS as well.

In this study, we investigated whether a lack of the MR in myeloid cells had any impact on CNS autoimmunity. We extended previous findings on the phenotypic changes of MR-deficient myeloid cells and additionally examined the functional consequences of MR deficiency for phagocytosis, the cells’ migratory behavior and their cytotoxic mechanisms. In essence, we found that mice with a myeloid cell-specific MR deficiency were partially protected from EAE due to an altered polarization of macrophages, monocytes, and microglia as well as indirect effects on T cells, supporting an important role of myeloid cells as target for GC in MS.

## Materials and Methods

### Animal Experimentation

Wild-type (wt) C57Bl/6 mice were purchased from Charles River (Sulzfeld, Germany) while the following mutant mouse strains (all on a C57BL/6 background) were bred in our animal facility in Göttingen: *Nr3c2^tm2Gsc^* (19), here designated MR^flox^; *Nr3c2^tm2Gsc^Lyz2tm1(cre)lfo/J* (26), here designated MR^lysM^; *Gt(ROSA)26Sor^tm1Hjf^(Tcra2D2,Tcrb2D2)1Kuch/J* (27), here designated RFP 2D2; and *Lyz2tm1(cre)lfo/J*, here designated LysM^cre^. All animal experiments were performed in accordance with the ethical standards of animal welfare and approved by the responsible German authorities in Lower Saxony (*LAVES*).

### EAE Induction

C57Bl/6 mice of the different genotypes were immunized with 50 µg myelin oligodendrocyte glycoprotein peptide 35–55 (MOG_35–55_) in CFA and treated twice with 400 ng pertussis toxin in total as described (12). Animals were weighed daily and scored for clinical signs of the disease on a scale from 0 to 10 depending on severity; scores were as follows: 0 = normal; 1 = reduced tone of tail; 2 = limp tail, impaired righting; 3 = absent righting; 4 = gait ataxia; 5 = mild paraparesis of hindlimbs; 6 = moderate paraparesis; 7 = severe paraparesis or paraplegia; 8 = tetraparesis; 9 = moribund; 10 = death.

### Immunohistochemistry and Histology

Analysis of PFA-fixed and paraffin-embedded spinal cord sections was performed according to standard protocols. In brief, 3 µm cross-sections were stained with an anti-human CD3 antibody (1:200; Serotec, Düsseldorf, Germany) or an anti-mouse MAC3 antibody (1:200; BD Biosciences, Heidelberg, Germany) followed by incubation with a biotinylated rabbit anti-rat antibody (1:200; Vector Laboratories, Burlingame, CA, USA). Antigen retrieval was achieved by pre-treating the sections in citrate buffer, pH = 6.0, for 20 min in a microwave oven at 850 W. 3,3′-diaminobenzidine (DAB) was used for visualization.

Myelin was stained by incubation of spinal cord sections in 0.1% LFB solution according to standard protocols. Slides were washed with 96% ethanol and ddH_2_O, and immersed in Li_2_CO_3_ for 30 s. Following washing with 70% ethanol, sections were incubated in 0.8% periodic acid for 10 min, washed with ddH_2_O, and subsequently incubated in Schiff’s reagent for 20 min. Axonal fibers were stained using silver impregnation (Bielschowsky technique) as previously described (28). Spinal cord sections were immersed in a 20% AgNO_3_ solution for 15 min, collected in ddH_2_O, and incubated in an AgOH solution for 20 min. After washing with 0.1% NH_3_, the sections were developed using a mixture of 25 ml AgOH and 50 µl of developer (37% formaldehyde, 63% nitric acid/citric acid). The slides were washed with 0.1% NH_3_ and ddH_2_O, and incubated for 5 min in sodium thiosulfate at 4°C to preserve the staining.

Stainings were quantified by taking pictures of five sections from each mouse using an Olympus BX51 microscope at 20× magnification. For each section, six photomicrographs that covered the entire area of the spinal cord were analyzed using ImageJ either by counting individual cells or measuring the affected tissue areas (http://rsb.info.nih.gov/ij/). For calculation of the axonal density, the axonal density outside the lesion in the normal appearing white matter was set to 100% and the axonal density in the lesions calculated accordingly.

### Flow Cytometry

Single-cell suspensions from spleen and lymph nodes were prepared using a 40 µm nylon mesh. Blood was collected in Alsevers and subjected to erythrolysis. CNS-infiltrating cells and sorted microglia and T cells were purified as described below. All antibodies were obtained from BioLegend (Uithoorn, The Netherlands) unless otherwise indicated and directly conjugated to a fluorochrome: anti-CD3ε (17A2), anti-TCRβ (H57-597), anti-CD4 (RM4-5, BD Biosciences), anti-CD11b (M1/70), anti-CD25 (PC61), anti-CD45.2 (104), anti-Ly6C (HK1.4), anti-Ly6G (1A8), and anti-FoxP3 (FJK-16s). Stainings were performed as previously described (12). Analysis was carried out using a FACSCanto II device (BD Biosciences) in combination with FlowJo software (Treestar, Ashland, OR, USA).

### Isolation of Cells from the Spinal Cord

Total leukocytes, microglia, and T cells were isolated from the spinal cord by density centrifugation following perfusion of the mice with NaCl as described (12). In brief, the dissected tissue was homogenized and mononuclear cells were separated in a three-layer Percoll gradient. After centrifugation, the cells were harvested at the interphases between the layers, washed with PBS and analyzed by flow cytometry. Microglia defined as CD45^int^CD11b^int^Ly6G^neg^CD3^neg^ as well as T cells defined as TCRβ^+^CD45^+^CD11b^neg^ were sorted using a FACSAria sorp device (BD Biosciences).

### T and B Cell Purification

Single-cell suspensions were prepared from lymph nodes and spleens. After erythrocyte lysis using ACK puffer (0.15 M ammonium chloride, 1 mM potassium hydrogene carbonate, and 0.1 mM sodium EDTA), T cells or B cells were purified using the EasySep™ Mouse T cell or B cell Enrichment Kit (Stemcell Technologies, Cologne, Germany), respectively, as previously described (29). The purity of the preparations was routinely greater than 95% as determined by flow cytometry.

### Macrophage Isolation and Culture

Bone marrow-derived macrophages (BMDM) were generated by culturing bone marrow cells obtained from femura and tibiae for 7 days in the presence L929-conditioned medium (LCCM) as described previously (30).

Peritoneal macrophages (PM) were elicited by injecting 1 ml 4% thioglycolate solution i.p. per mouse 4 days prior to their isolation, and obtained *via* peritoneal lavage using PBS with 0.1% BSA. The cells were seeded in 10 cm plates in DMEM medium with 10% FCS and incubated for 2 h. Non-adherent cells were removed by repeated washings with PBS with 0.1% BSA and the adhered macrophages were detached by incubation with 2 mM EDTA in PBS for 20 min. Purity of the preparations was usually around 90% as determined by flow cytometry based on CD11b staining.

### *In Vitro* Analysis of Macrophage Functions

Bone marrow-derived macrophages were cultured for 24 h in DMEM medium with 10% FCS supplemented with 20 ng/ml LPS and 50 ng/ml IFNγ. NO concentrations in BMDM culture supernatants were determined using Griess reagent as described previously (15), cytokine levels were measured using commercially available ELISA kits for IL-6 and TNFα (BioLegend) according to the manufacturer’s instructions.

For the analysis of phagocytosis, lymph node cells from C57Bl/6 wt mice were labeled with CFSE as described below and irradiated for 6 min with a dose of 5 Gy/min to induce apoptosis. The irradiated lymphocytes were incubated in DMEM medium with BMDM or PM at a ratio of 1:2 for 2 or 20 h, and CFSE fluorescence within the macrophage populations determined by flow cytometry. *In vitro* migration assays of PM were performed essentially as recently described (16).

### *In Vitro* Activation of MOG-Specific T Cells

T cells obtained from RFP 2D2 mice were labeled with CFSE in order to be able to monitor their proliferation. Cell suspensions were adjusted to 2 × 10^6^ cells/ml in PBS followed by an incubation at 37°C with 0.25 µM CFSE for 10 min. The reaction was stopped by adding FCS to a final concentration of 2%. Cells were washed with PBS and re-suspended in DMEM medium with 10% FCS. 1 × 10^5^ CSFE-labeled T cells were co-cultured with 1 × 10^5^ bone marrow-derived macrophages (BMDM) in the presence of 20 µM MOG_35–55_. Cultures were harvested after 48 and 72 h and proliferation was assessed by flow cytometry. Alternatively, RFP^+^ Th17-polarized MOG-specific T cells were cultured with BMDM and MOG_35–55_ for 72 h and the level of IL-17A was determined in the supernatant by ELISA using a commercially available kit (BioLegend).

### *In Vivo* Proliferation Assay

T cells were purified from RFP 2D2 mice and labeled with CFSE. Subsequently, 1 × 10^6^ of the cells were injected i.v. into mice. Two days later, the mice were immunized according to our standard protocol. On days 3 and 5, mice were sacrificed, spleen and lymph nodes dissected, and proliferation assessed by flow cytometry.

### Re-Stimulation Assay and Cytokine ELISA

6 × 10^5^ splenocytes or 3 × 10^5^ lymph node cells were isolated from mice at day 10 after immunization and seeded in 96 well-plates. The cells were incubated for 72 h in DMEM medium with 5% FCS in the presence of 20 µg/ml of MOG_35–55_ peptide. Cytokine levels in the culture medium were determined using commercially available ELISA kits for IFNγ, TNFα, IL-17A (BioLegend), or GM-CSF (R&D Systems, Wiesbaden, Germany) according to the manufacturer’s instructions.

### Western Blot

Lysates were prepared from BMDM in denaturating sample buffer containing a protease inhibitor cocktail (RIPA with NP40, NA_3_VO_4_, and Na_3_MoO_4_) and heated at 95°C for 5 min (22). 15 µg proteins were separated on a 7.5% SDS-PAGE gel, transferred to a nitrocellulose membrane (Amersham, Braunschweig, Germany) and stained with a rabbit anti-MR or a rabbit anti-ERK antibody (both 1:1,000, Santa Cruz, Heidelberg, Germany). Visualization was achieved using ECL substrate (Roth, Karlsruhe, Germany) in combination with a ChemiLux Imager (Intas, Göttingen, Germany).

### RNA Isolation and Quantitative RT-PCR

Total RNA from macrophages, T and B cells, CNS-infiltrating lymphocytes, or microglia was isolated using the Quick-RNA MiniPrep kit (Zymo Research, Irvine CA, USA) and reverse transcribed into cDNA with the help of the iScript kit (Bio-Rad, Munich, Germany). Quantitative RT-PCR was performed on an ABI 7500 instrument (Applied Biosystems, Darmstadt, Germany) using SYBR green mastermix from the same company according to the manufacturer’s instruction. The results were normalized to the mRNA expression of HPRT serving as a housekeeping gene and evaluated with the ΔΔCt method. Primer sequences are available upon request.

### Analysis of Sodium and Chloride Concentrations in the Serum

Serum was collected from MR^flox^ and MR^lysM^ mice by heart puncture and centrifugation. Subsequently, it was analyzed on an architect c system using an ICT (Na^+^, K^+^, Cl^−^) sample dilution kit (Abbott Laboratories, Hannover, Germany).

### Statistical Analysis

Analyses of EAE experiments were performed using the Mann–Whitney *U* test. All other analyses were performed by unpaired *t*-test. Data are depicted as mean ± SEM; *p* values above 0.05 were considered as non-significant (n.s.); *p < 0.05, **p < 0.01, ***p < 0.001.

## Results

### Phenotypic and Functional Changes in MR-Deficient Macrophages

**Figure 1:**
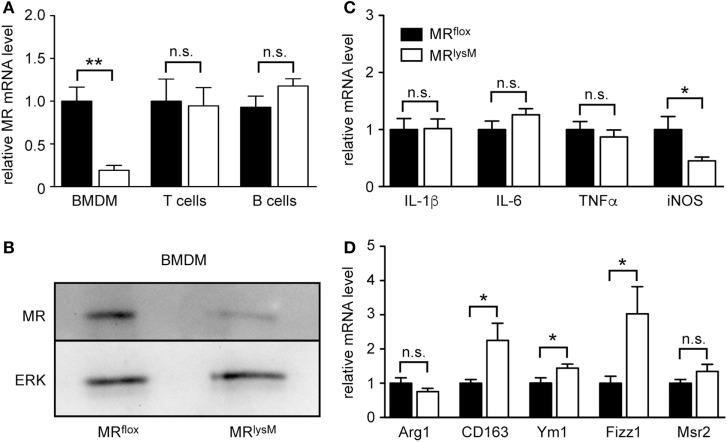
Phenotypic characterization of bone marrow-derived macrophages (BMDM) from MR^lysM^ mice. **(A)** Unstimulated BMDM as well as magnetically sorted T and B cells from the lymph nodes of MR^lysM^ mice in comparison to MR^flox^ littermate controls were analyzed for gene expression levels of the mineralocorticoid receptor (MR). *n* = 5 (BMDM), *n* = 3 (T and B cells). **(B)** Protein levels of the MR were studied by western blot analysis of whole protein extracts made from unstimulated BMDM of MR^lysM^ or MR^flox^ origin. An antibody to ERK was used as a loading control. One representative example out of three is depicted. **(C,D)** BMDM were stimulated with LPS/IFNγ and analyzed for mRNA expression levels of genes being typical either for an M1 **(C)** or M2 **(D)** polarization. *n* = 18. Black bars refer to BMDM from MR^flox^ mice and white bars to BMDM from MR^lysM^ mice. All data are presented as the mean ± SEM (n.s., not significant, *p < 0.05, **p < 0.01).

Initially, we aimed to verify an efficient and specific deletion of the MR in myeloid cell-specific MR knock-out mice. BMDM were differentiated from the bone marrow of MR^flox^ and MR^lysM^ mice and analyzed by quantitative RT-PCR and western blot. Gene expression of the MR was reduced by about 80% in BMDM from MR^lysM^ mice (Figure 1A) and MR protein levels were strongly diminished as well (Figure 1B). By contrast, MR mRNA expression in purified T and B cells from the lymph nodes of MR^lysM^ and MR^flox^ mice was similar (Figure 1A), confirming the cell-type specificity of the mutation. Considering that the MR plays a central role in the regulation of the salt-water balance, we analyzed sodium and chloride concentrations in the serum. However, there was no difference between mice of both genotypes (MR^flox^: 150 ± 4 mM Na^+^, 118 ± 1 mM Cl^−^; MR^lysM^: 150 ± 3 mM Na^+^, 115 ± 3 mM Cl^−^; *n* = 5/7).

**Figure 2:**
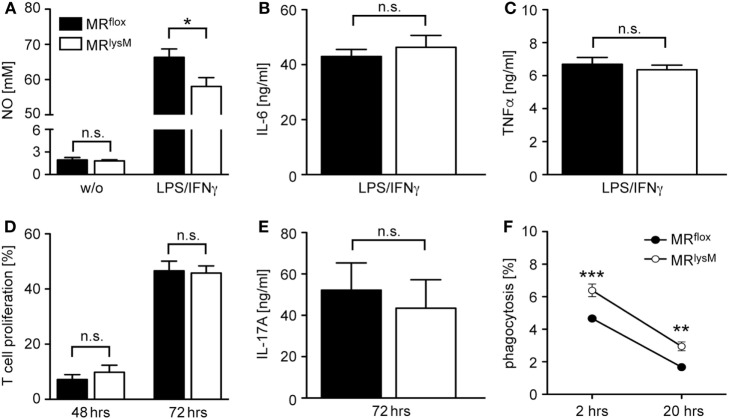
Functional characterization of bone marrow-derived macrophages (BMDM) from MR^lysM^ mice. **(A)** Production of NO by unstimulated (w/o) or LPS/IFNγ-stimulated BMDM from MR^lysM^ or MR^flox^ mice after 24 h *in vitro* culture. *n* = 9–11. **(B,C)** Production of IL-6 or TNFα by LPS/IFNγ-stimulated BMDM from MR^lysM^ or MR^flox^ mice after 24 h *in vitro* culture. *n* = 16–24. **(D)** *In vitro* proliferation of CFSE-labeled RFP^+^ 2D2 T cells stimulated with their cognate antigen in the presence of BMDM from MR^lysM^ or MR^flox^ mice after 48 and 72 h. T cells with diluted CFSE were considered as proliferative regardless of their round of cell division. *n* = 9. **(E)** IL-17A production by Th17-differentiated 2D2 cells cultured with BMDM from MR^lysM^ or MR^flox^ mice after 72 h. *n* = 11. **(F)** Analysis of the phagocytic activity of BMDM by incubating them with CFSE-labeled irradiated lymph node cells from C57BL/6 wt mice at a 2:1 ratio. The frequency of CFSE^+^ BMDM was measured by flow cytometry after 2 h and 20 h. *n* = 10–12. Black bars/symbols refer to BMDM from MR^flox^ mice and white bars/symbols to BMDM from MR^lysM^ mice. All data are presented as the mean ± SEM (n.s., not significant, *p < 0.05, **p < 0.01, ***p < 0.001).

Since it was reported that deletion of the MR in myeloid cells changes their phenotype (9), we next investigated the expression of genes associated with macrophage polarization. The mRNA levels of the pro-inflammatory cytokines IL-1β, IL-6, and TNFα in BMDM from MR^lysM^ mice stimulated with LPS and IFNγ were comparable to those in BMDM from MR^flox^ mice (Figure 1C). By contrast, we observed a downregulation of iNOS, an enzyme being involved in NO metabolism (Figure 1C), and an upregulation of several genes characteristic for an M2 polarization (Figure 1D). Furthermore, NO production by MR-deficient BMDM was diminished in response to LPS/IFNγ stimulation (Figure 2A), pointing toward a reduced cytotoxicity. Consistent with the observed gene expression profile (see Figure 1C), there was no change in the production of the pro-inflammatory cytokines IL-6 and TNFα after stimulating BMDM with LPS/IFNγ (Figures 2B,C). Similarly, the capacity of BMDM to activate T cells *in vitro* as assessed using CFSF-labeled T cells from 2D2 mice expressing a MOG-specific TCR was unaffected by the deletion of the MR in myeloid cells. Namely, T-cell proliferation in the presence of antigen was the same regardless of the genotype of the BMDM (Figure 2D) as it was the case for IL-17A production by Th17-polarized 2D2 T cells (Figure 2E). By contrast, the ability of MR-deficient BMDM to phagocytose apoptotic lymph node cells was enhanced in comparison to MR^flox^ control cells (Figure 2F).

**Figure 3:**
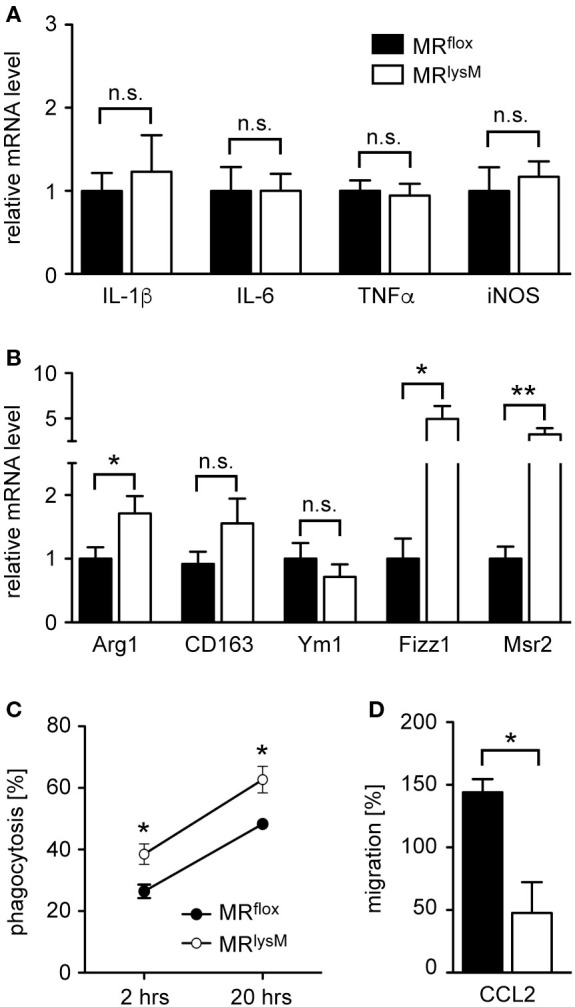
Phenotypic and functional characterization of peritoneal macrophages (PM) from MR^lysM^ mice. **(A,B)** PM were analyzed for mRNA expression levels of genes being typical for an M1 **(A)** or M2 **(B)** polarization. *n* = 20. **(C)** Analysis of the phagocytic activity of PM by incubating them with CFSE-labeled irradiated lymph node cells from C57BL/6 wt mice at a 2:1 ratio. The frequency of CFSE^+^ PM was measured by flow cytometry after 2 and 20 h. *n* = 4–6. **(D)** The migratory capacity of PM was determined by culturing them in a transwell system with a gradient of 50 ng/ml CCL2. The transmigrated cells in the lower chamber were quantified by FACS analysis and their number after spontaneous migration without CCL2 was set to 100%. *n* = 4. Black bars/symbols refer to PM from MR^flox^ mice and white bars/symbols to PM from MR^lysM^ mice. All data are presented as the mean ± SEM (n.s., not significant, *p < 0.05, **p < 0.01).

Next, we investigated thioglycolate-elicited PM as an example of a more mature developmental stage of myeloid cells. In accordance with our findings for BMDM (see Figures 1C,D), we observed unaltered mRNA levels for several M1 genes while the majority of markers associated with an M2 polarization were upregulated (Figures 3A,B). This phenotypic change was accompanied by an enhanced phagocytosis rate of PM derived from MR^lysM^ mice in comparison to MR^flox^ mice (Figure 3C) and a diminished migratory capacity of MR-deficient PM toward the chemokine CCL2 (Figure 3D). In summary, a deletion of the MR in macrophages alters their phenotype based on gene expression, which in turn has consequences for cytotoxicity, phagocytosis, and migration.

### MR^lysM^ Mice Show Reduced Neuroinflammation in an EAE Model

**Figure 4:**
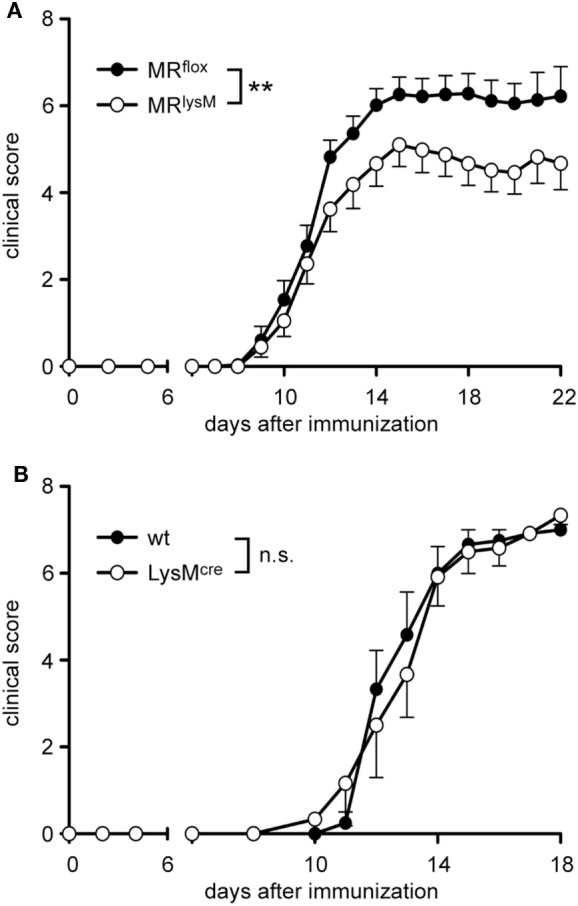
Clinical experimental autoimmune encephalomyelitis (EAE) score of MR^lysM^ and LysM^cre^ mice. EAE was induced by immunization with MOG_35–55_ in CFA. **(A)** Disease course of MR^flox^ and MR^lysM^ mice. *n* = 25/29, pooled from three individual experiments. **(B)** The disease course of wild-type and LysM^cre^ mice was monitored over a period of 18 days. *n* = 6/6. Data are presented as mean ± SEM (n.s., not significant, **p < 0.01).

It was previously shown that monocytes and macrophages play an important role in the maintenance and effector phase of EAE (31). Therefore, we investigated whether the altered phenotype of myeloid cells in MR^lysM^ mice had any consequences in the MOG_35–55_ induced EAE model. The onset of the disease in MR^lysM^ mice compared to MR^flox^ controls was similar but the severity in the chronic phase was significantly reduced in the mutant mice (Figure 4A). The observed difference in the disease course cannot be attributed to the cre recombinase inserted into the lysosyme M locus in MR^lysM^ mice, since EAE development was similar in wt and LysM^cre^ C57Bl/6 mice (Figure 4B).

**Figure 5:**
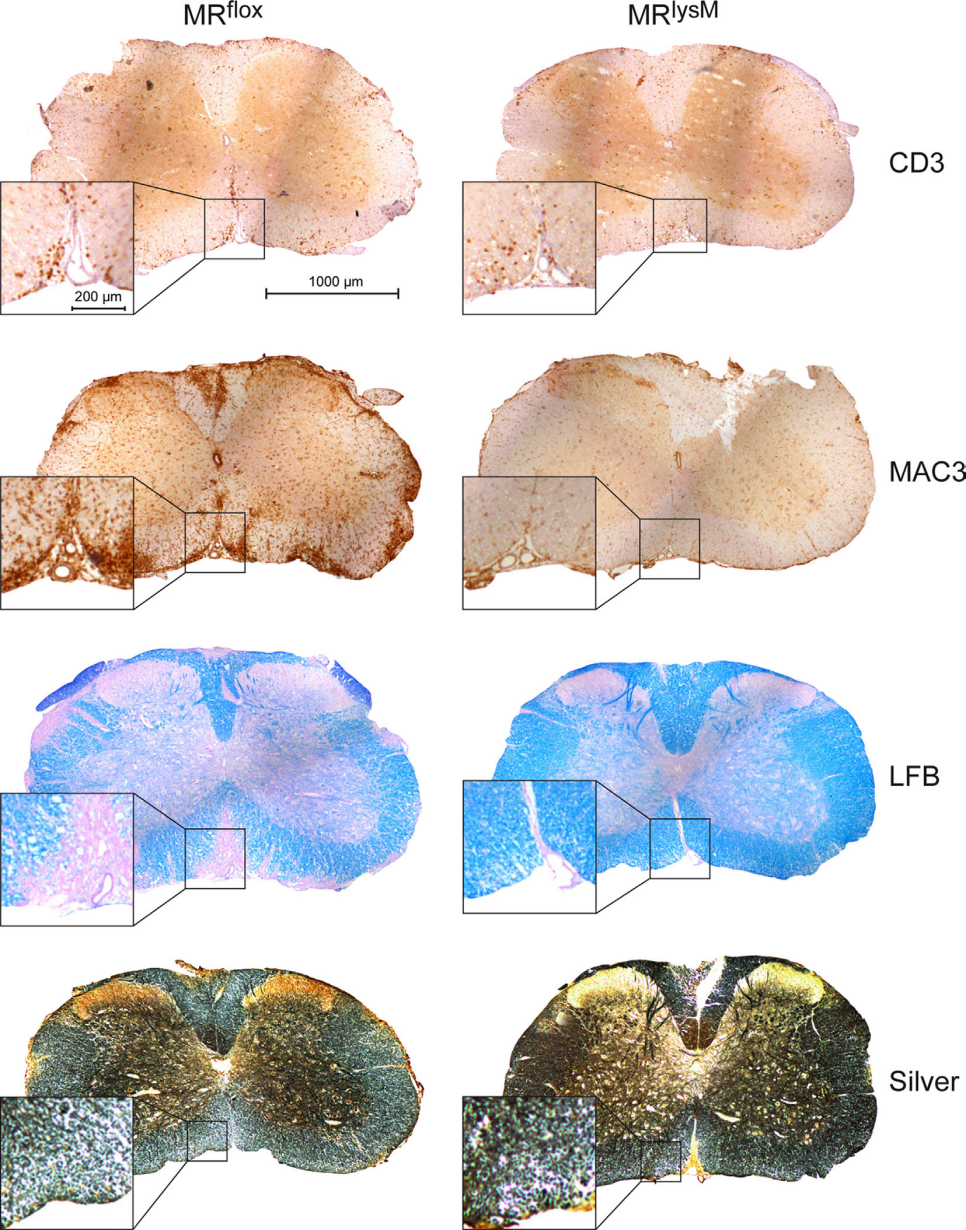
Immunohistochemical and histological evaluation of neuroinflammation at the peak of disease in MR^flox^ and MR^lysM^ mice (about day 14 p.i.). The pictures show one representative cross section for each staining for MR^flox^ (left) and MR^lysM^ mice (right) covering the entire spinal cord and additionally a higher magnification area. The sections were incubated with antibodies recognizing CD3 or MAC3, stained with LFB to visualize demyelination or by using the Bielschowsky silver impregnation method to reveal axonal density (from top to bottom). Scale bars are 1,000 and 200 µm, respectively.

**Figure 6:**
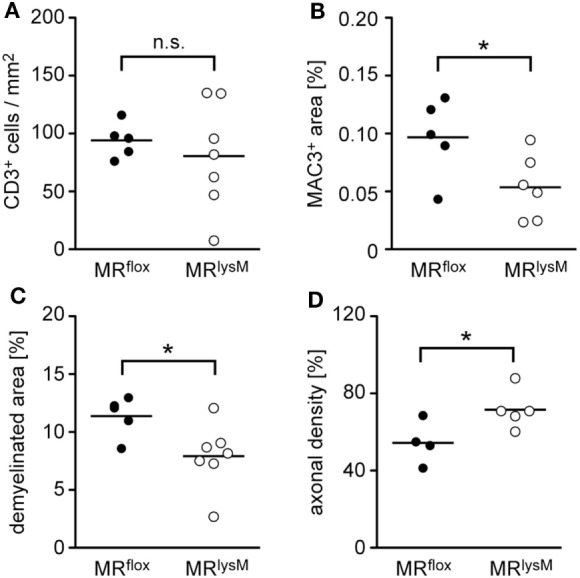
Quantification of immunohistochemical and histological hallmarks of neuroinflammation at the peak of disease in MR^flox^ and MR^lysM^ mice (about day 14 p.i.). Infiltration of CD3^+^ T cells **(A)** and MAC3^+^ macrophages/monocytes **(B)** was determined after staining the cells with the respective antibodies. Demyelination was assessed by LFB staining **(C)** and axonal density by Bielschowsky silver impregnation **(D)**. The axonal density in the normal appearing white matter outside the lesion was set as 100%. Every dot represents the data from one individual mouse. *n* = 4–7 (n.s., not significant, *p < 0.05).

To support our clinical finding, we performed a histopathological analysis of the spinal cord. T cell infiltration at the peak of disease was unaffected based on immunohistochemistry using an anti-CD3 antibody (Figures 5 and 6A), whereas numbers of myeloid cells positively staining for MAC3 were significantly reduced (Figures 5 and 6B). Consistent with the lower clinical score, there was less demyelination and a higher axonal density in MR^lysM^ compared to MR^flox^ mice, indicating improved neuronal preservation (Figures 5 and 6C,D).

### T-Cell Differentiation in Peripheral Lymphoid Organs Is Impaired in MR^lysM^ Mice

**Figure 7:**
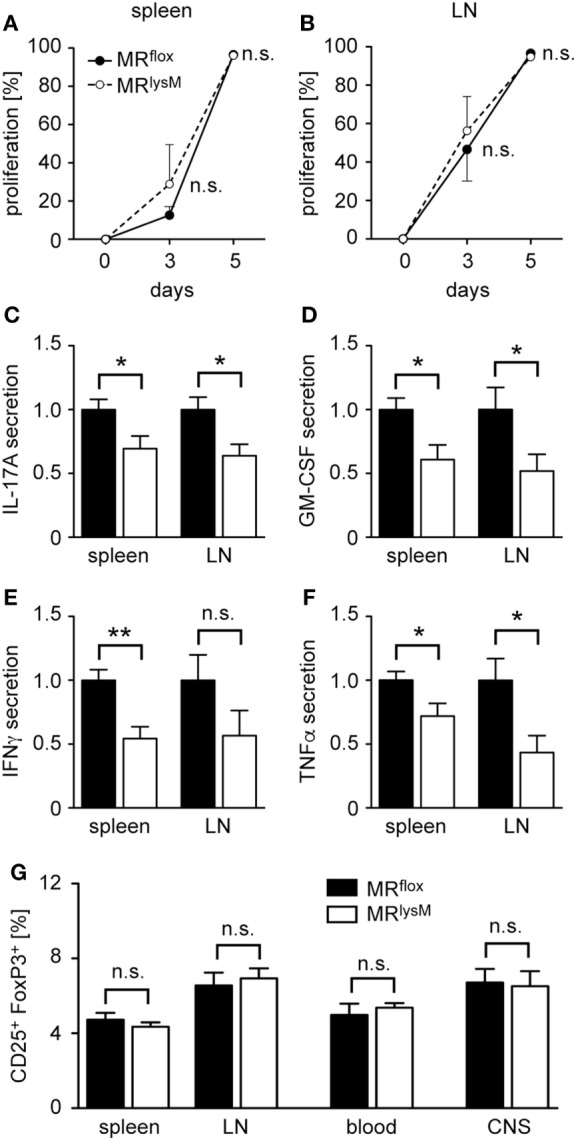
T cell proliferation and cytokine production in peripheral lymphoid organs and regulatory T cell (Treg) abundance after experimental autoimmune encephalomyelitis induction in MR^lysM^ mice. **(A,B)** MR^flox^ and MR^lysM^ mice were adoptively transferred with CFSE-labeled RFP^+^ 2D2 T cells and subsequently immunized with MOG_35–55_ in CFA. Proliferation of MOG-specific T cells was investigated at days 3 and 5 p.i. in spleen **(A)** and draining lymph nodes **(B)** by FACS analysis. T cells with diluted CFSE were considered as proliferative regardless of their round of cell division. *n* = 3. **(C–F)** Production of the pro-inflammatory cytokines IL-17A **(C)**, GM-CSF **(D)**, IFNγ **(E)**, and TNFα **(F)** *in vitro* by splenocytes and lymph node cells at day 10 p.i. and after an additional culture for 72 h in the presence of the cognate antigen. *n* = 5–10. **(G)** Frequency of CD25^+^FoxP3^+^ Treg in spleen, draining lymph nodes, blood and spinal cord [central nervous system (CNS)] as measured by FACS analysis at day 10 p.i. for peripheral organs and at the peak of disease for spinal cord. *n* = 14–17 (peripheral organs), *n* = 11 (spinal cord). Black bars/symbols refer to values from MR^flox^ mice and white bars/symbols to values from MR^lysM^ mice. All data are presented as the mean ± SEM (n.s., not significant, *p < 0.05, **p < 0.01).

To elucidate the mechanisms being responsible for the diminished neuroinflammation in myeloid cell-specific MR knock-out mice, we analyzed whether the M2 phenotype of mutant macrophages had any impact on T-cell priming after immunization. To this end, MR^lysM^ and MR^flox^ mice received CFSE-labeled MOG-specific T cells from RFP 2D2 mice, which allowed us to monitor their expansion in the host and which revealed that T-cell proliferation in spleen and lymph nodes was unaffected by the absence of the MR in myeloid cells at days 3 and 5 after immunization (Figures 7A,B). To investigate cytokine secretion, T cells from MR^flox^ and MR^lysM^ mice were isolated at day 10 after immunization, i.e., shortly before the onset of clinical symptoms, and re-stimulated with their cognate antigen *in vitro*. Despite the unaltered T-cell proliferation, the production of major pro-inflammatory cytokines, such as IFNγ, IL-17A, GM-CSF, and TNFα, by splenocytes and lymph node cells was significantly reduced in mice lacking the MR in their myeloid cells (Figures 7C–F). Finally, we asked whether the impaired effector T-cell differentiation might be linked to changes in the frequency of CD25^+^FoxP3^+^ regulatory T cells (Tregs). However, we did not observe any differences in their percentages at day 10 after immunization in any lymphoid compartment when comparing MR^lysM^ and MR^flox^ mice (Figure 7G). Hence, different numbers of Treg were not responsible for the reduced production of pro-inflammatory cytokines by antigen-specific T cells in MR^lysM^ mice.

### The Frequency of Inflammatory Monocytes and Reactive Microglia Is Reduced in the CNS of MR^lysM^ Mice

**Figure 8:**
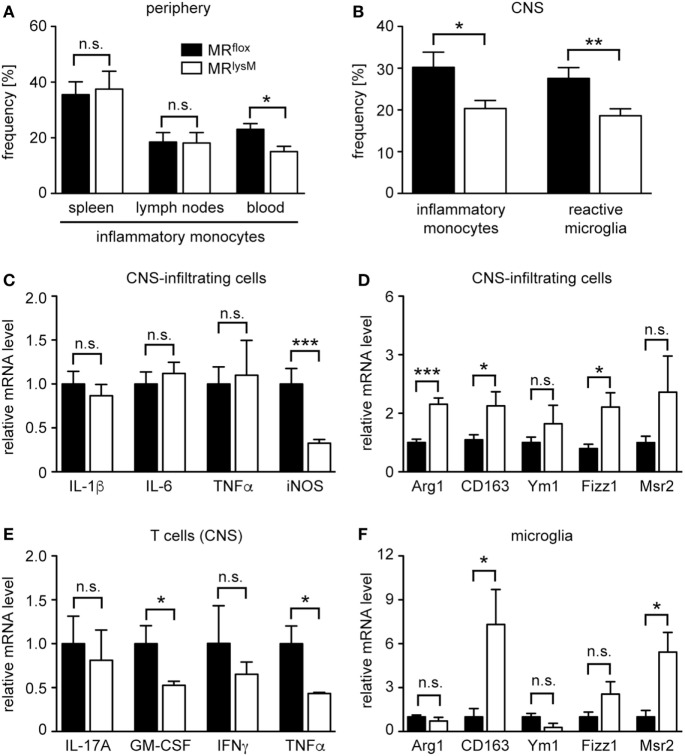
Characterization of the phenotype and abundance of monocytes, microglia, and central nervous system (CNS)-infiltrating cells in MR^lysM^ mice. **(A)** Frequency of inflammatory CD11b^+^Ly6G^−^Ly6C^high^ monocytes in spleen, lymph nodes, and blood of MR^flox^ and MR^lysM^ mice 10 days after experimental autoimmune encephalomyelitis induction as assessed by FACS analysis. *n* = 7/9 (secondary lymphoid organs), *n* = 4/5 (blood). **(B)** Frequency of inflammatory monocytes (here defined as CD45^high^CD11b^high^Ly6C^high^) and reactive microglia (here defined as CD45^int^CD11b^int^Ly6C^high^) in the CNS as assessed by FACS analysis of the spinal cord at the peak of disease in MR^lysM^ or MR^flox^ mice. *n* = 14–16 (monocytes), *n* = 18–21 (microglia). **(C,D)** CNS-infiltrating cells isolated from the spinal cord of MR^lysM^ or MR^flox^ mice at the peak of disease were analyzed for mRNA expression levels of genes being typical either for an M1 **(C)** or M2 **(D)** polarization. *n* = 16–18. **(E)** T cells were FACS-sorted from the spinal cord of MR^lysM^ or MR^flox^ mice at the peak of disease and analyzed for gene expression of pro-inflammatory cytokines. *n* = 3/4. **(F)** Microglia from MR^lysM^ or MR^flox^ mice were FACS-sorted from the spinal cord at the peak of disease and analyzed for mRNA expression levels of genes being typical for an M2 polarization. *n* = 6/7. Black bars refer to values from MR^flox^ mice and white bars to values from MR^lysM^ mice. All data are presented as the mean ± SEM (n.s., not significant, *p < 0.05, **p < 0.01, ***p < 0.001).

Following EAE induction, monocytes migrate to the CNS where they differentiate *in situ* into macrophages. As the frequency of monocytes with an inflammatory phenotype is known to impact disease severity, we studied whether this feature was altered in mice lacking the MR in myeloid cells. CD11b^+^Ly6G^−^ monocytes can be divided into cells with an inflammatory and a resting phenotype based on Ly6C surface expression (32). Interestingly, there was no difference in the percentage of inflammatory monocytes in secondary lymphoid organs, such as spleen and lymph nodes 10 days after EAE induction (Figure 8A). By contrast, there were fewer inflammatory Ly6C^high^ monocytes circulating in the blood of MR^lysM^ mice than was the case in control animals (Figure 8A). The observed difference for inflammatory monocytes was even stronger in the CNS, here defined as CD45^high^CD11b^high^Ly6C^high^, where we observed a markedly decreased frequency of this cell type in MR^lysM^ mice compared to MR^flox^ controls (Figure 8B). Of note, the frequency of Treg in the CNS was similar in both genotypes (Figure 7G).

Next, we studied gene expression in CNS-infiltrating cells. In line with our analysis of BMDM and PM (see Figures 1 and 3), we observed unaltered mRNA levels of IL-1β, IL-6, and TNFα, genes that are typical for an M1 phenotype, a downregulation of iNOS, and an upregulation of several genes associated with an M2 polarization in MR^lysM^ mice (Figures 8C,D). Since we found that peripheral T cells from immunized MR^lysM^ mice produced less pro-inflammatory cytokines compared to MR^flox^ mice, we tested whether this was also the case for T cells that had migrated into the CNS during EAE. When we purified infiltrating T cells from the spinal cord, it turned out that gene expression of IL-17A, GM-CSF, IFNγ, and TNFα was either downregulated or unchanged in MR^lysM^ knock-out mice (Figure 8E). We conclude that a lack of the MR in myeloid cells reduces the inflammatory milieu in the CNS of EAE mice by affecting both the polarization of macrophages/monocytes and indirectly the activation state of T cells.

### The Phenotype of the Microglia Is Altered in MR^lysM^ Mice during EAE

Microglia serve as local APC in the CNS and are implicated in the development of EAE (33, 34). As our previous analysis had indicated that a cre recombinase expressed under the control of the LysM promotor allows for efficient recombination of floxed genes in microglia briefly after birth (15), we also studied the phenotype of this cell type during EAE. The frequency of reactive microglia, here defined as CD45^int^CD11b^int^Ly6C^high^, was diminished in MR^lysM^ mice at the peak of disease compared to MR^flox^ littermate controls (Figure 8B), indicating that there was a shift from a pro-inflammatory to an anti-inflammatory phenotype in this compartment. To support this notion, we isolated microglia from the CNS of MR^lysM^ and MR^flox^ mice at the peak of EAE by preparative flow cytometry and analyzed them by quantitative RT-PCR. Upregulation of CD163 and Msr2 mRNA levels confirmed that microglia were committed to an M2 phenotype in MR^lysM^ mice (Figure 8F).

## Discussion

Glucocorticoids bind to the MR with equal affinity as aldosterone. Therefore, in cell types which do not express the GC-inactivating enzyme 11β-HSD2, including myeloid cells, GC may not only act *via* the GR but alternatively exert their function *via* the MR. It has been noted that activation of the MR by specific agonists including GC induces a pro-inflammatory phenotype in myeloid cells (9, 35), and it has been shown that PM lacking the MR have a different transcriptional profile resembling M2-polarized macrophages (9, 23, 36). Mice deficient for the MR specifically in myeloid cells were protected against cardiac hypertrophy, fibrosis, and vascular damage *in vivo* (9, 36) and showed a reduced infarct volume and less cerebral inflammation in a model of stroke (23). In this study, we extended these findings by showing that also functional features of macrophages such as their phagocytic and migratory activity and their cytotoxicity were influenced by a lack of the MR. Furthermore, we found that mice with a myeloid cell-specific deficiency of the MR presented with milder neuroinflammation in an EAE model, which could be explained by a number of immunological alterations present in these mice. Our findings are in line with a previous report demonstrating that application of an MR agonist aggravated clinical symptoms of EAE, which could be prevented by spironolactone (17).

First, myeloid cells in MR^lysM^ mice have a less inflammatory phenotype in the blood and CNS. It is well known that myeloid cells, especially activated monocytes and macrophages, are recruited in high numbers to the inflamed CNS during EAE, where they even outnumber encephalitogenic T cells. On the one hand, myeloid cells are considered to be major effector cells responsible for myelin damage and axon destruction (37), for instance, by producing NO and ROS (38). On the other hand, macrophages with phagocytosed myelin can be detected in lesions in MS and EAE where they are also thought to contribute to tissue repair (39, 40). It was found that EAE was ameliorated when macrophages were depleted using dichloromethylene diphosphonate-containing liposomes (41, 42), when the migration of monocytes/macrophages into the CNS was prevented by deletion of CCR2 (31), or when alternatively activated M2 macrophages were administered (43, 44). We could further show that the M2 polarization of myeloid cells using GC encapsulated in liposomes or inorganic–organic hybrid nanoparticles improved clinical symptoms of EAE (15, 16). Recently, it was reported that activated invading monocyte-derived macrophages initiate demyelination whereas microglia rather appear to clear cellular debris (45). Other authors, however, reported detrimental effects of M1-polarized microglia (46). In the study at hand, we found that microglia cells in MR^lysM^ mice were polarized toward a non-inflammatory resting phenotype, which potentially could contribute to the ameliorated EAE disease course. As pointed out earlier, the MR in myeloid cells is mainly bound by GC and modulates the function of macrophages and microglia cells in a balanced manner in cooperation with the GR (8, 10, 47). For this reason, we believe that effects caused by MR deletion in myeloid cells can be exclusively attributed to the activity of endogenous GC while aldosterone does not play a role.

Second, the differentiation of antigen-specific T cells in the periphery was impaired in MR^lysM^ mice. Although we did not notice a generally reduced antigen-presenting capacity of myeloid cells as exemplified by the analysis of BMDM, the secretion of several pro-inflammatory cytokines by antigen-specific T cells was significantly diminished at day 10 after immunization, i.e., shortly before clinical onset. By contrast, T-cell proliferation and the number of Treg were unchanged in mutant mice. Interestingly, gene expression of pro-inflammatory cytokines by T cells that migrated into the CNS was also diminished in MR^lysM^ mice, suggesting that the deactivated state of peripheral T cells in mutant mice persists after infiltration into the spinal cord. There is experimental evidence for a contribution of Th1 and Th17 cells in the pathogenesis of EAE (48–52). Major cytokines produced by Th1 and Th17 cells were reduced in MR^lysM^ mice, pointing toward a rather general mode of action concerning cytokine suppression rather than a specific one affecting only one specific Th cell subset. Treg are potential candidates for suppressing immune responses in a more general way, but as the frequency of Treg was unaffected both in the periphery and the CNS, this possibility seems rather unlikely. Instead, alternatively activated macrophages with an M2 phenotype might explain the observed effects as they were previously associated with diminished T-cell activation, proliferation, and cytokine production. For instance, increased *arg1* expression was shown to result in a decrease in l-arginine levels, a loss in CD3ζ by activated T cells and eventually in an impaired T-cell activation (53, 54). It is tempting to speculate that the shift toward the M2 phenotype of myeloid cells in MR^lysM^ mice results in a defective peripheral activation of encephalitogenic T cells *via* this pathway. Alternatively, re-activation of antigen-specific T cells within the CNS could also be affected because myeloid cells in the CNS and microglia adopted an M2 phenotype in MR^lysM^ mice. Whether the reduced expression of pro-inflammatory cytokines by T cells in the spinal cord is due to an impaired T-cell activation in the periphery or a dampened re-activation in the CNS remains to be further investigated.

Mineralocorticoid receptor-deficient myeloid cells adopt an M2 phenotype generally associated with the ability to decrease inflammation and support tissue repair. Overall, such a qualitative change was observed for all subtypes of myeloid cells regardless of their source and activation state. The phagocytic activity of BMDM and PM was increased in the absence of the MR, several M1 markers were either unchanged or reduced in all cell types analyzed, and mRNA levels of M2-specific genes were upregulated or unaltered. Nevertheless, there were also quantitative differences between cell populations. Expression of iNOS was diminished in LPS/IFNγ-stimulated BMDM and CNS-infiltrating cells, two examples for highly activated cell populations, whereas it was unaltered in PM lacking such a strong stimulus. Similarly, the expression profile of M2 markers differed between cell types. CD163 and Fizz1 were increased in 3 out of 4 cell types investigated, Arg1 and Msr2 in 2 out of 4, whereas Ym1 was increased only in LPS/IFNγ-stimulated BMDM. This observation could either reflect a functional diversity between myeloid subtypes or it could be explained by differences in the activation status, cellular source or pathological context. Taken together, our results confirm an important role of the MR in myeloid cell polarization.

There are conflicting data concerning the expression of the MR in lymphocytes with reports of low expression of the MR on human T cells (55) and negligible expression on purified mouse CD4^+^ and CD8^+^ T cells (17). Interestingly, we found a reasonable level of MR mRNA expression in T and B cells which, however, was unchanged in MR^lysM^ mice. While this observation confirms the cell type-specificity of the employed knock-out mice, it still raises the intriguing question as to the functional significance of the MR in leukocyte subpopulations other than macrophages and microglia in neuroinflammation. Since it was recently reported that the control of blood pressure and heart function was impaired in mice with a T cell-specific deletion of the MR (24, 25), we imagine that the MR in this cell type could also be involved in the control of neuroinflammatory diseases.

A major physiological role of the MR is the regulation of the salt-water balance by regulating sodium reabsorption in kidney and colon. Since it was demonstrated that sodium chloride impacts CNS autoimmunity by inducing Th17 cells and polarizing macrophages toward a pro-inflammatory phenotype (56–58), it was important to exclude that none of the effects observed in our study were due to an altered electrolyte homeostasis in MR^lysM^ mice. We found that both sodium and chloride levels in the serum were similar regardless of the genotype, reconfirming that expression of the cre recombinase in mutant mice was restricted to myeloid cells and did not affect regulation of the renin–angiotensin–aldosterone system (RAAS) as it is the case for mice in which the MR was ubiquitously inactivated (19, 22).

In summary, we provide evidence that the lack of the MR in myeloid cells impacts on the polarization of macrophages, thereby shaping their activity profile in response to endogenous GC. The phenotypic switch of myeloid cells, in concert with secondary changes in T-cell differentiation, results in diminished neuroinflammation after EAE induction, which identifies this pathway as a potential novel therapeutic target in MS. There are several MR antagonists already applied in the clinic, making this intracellular receptor a particularly promising target for pharmaceutical intervention. Spironolactone and eplerenone are both widely used to treat hypertension and heart failure with the latter being the less potent but more specific MR ligand (5, 59). More recently, a novel non-steroidal MR antagonist with improved potency and specificity for the MR has been developed (60, 61). Finerone, which is currently being tested in a phase II trial, provides protection against renal and cardiac insults and can be orally administered. As all of these MR antagonists are systemically active, potential adverse effect would need to be considered when being applied to interfere with neuroinflammatory diseases. Alternatively, novel drug delivery vehicles such as liposomes or nanoparticles could be used to specifically target MR antagonists to myeloid cells (15, 16). Thus, the combination of an efficient pharmacological blockade of the MR with state-of-the-art nanotechnology has the potential to improve MS therapy in the future.

## Ethics Statement

This study was carried out in accordance with the recommendations of the LAVES (Niedersächsisches Landesamt für Verbraucherschutz und Lebensmittelsicherheit).

## Author Contributions

EM-C: performed and analyzed most of the experiments, NS and XL: performed and analyzed experiments, HF: performed and analyzed experiments, critically revised text and figures, HR and FL: designed the project, analyzed experiments, and wrote the manuscript.

## Conflict of Interest Statement

The authors declare that the research was conducted in the absence of any commercial or financial relationships that could be construed as a potential conflict of interest.
